# Silica Aerogel Monoliths Derived from Silica Hydrosol with Various Surfactants

**DOI:** 10.3390/molecules23123192

**Published:** 2018-12-04

**Authors:** Dong Chen, Xiaodong Wang, Wenhui Ding, Wenbing Zou, Qiong Zhu, Jun Shen

**Affiliations:** School of Physics Science and Engineering & Shanghai Key Laboratory of Special Artificial Microstructure Materials and Technology, Tongji University, Shanghai 200092, China; Dongchen326@163.com (D.C.); Giacinta_hui@163.com (W.D.); zouwenbing@tongji.edu.cn (W.Z.); zhuqiong113@163.com (Q.Z.)

**Keywords:** silica hydrosol, aerogel, ambient pressure drying, surfactant

## Abstract

Owing to their ultra-low thermal conductivity, silica aerogels are promising thermal insulators; however, their extensive application is limited by their high production cost. Thus, scientists have started to explore low-cost and easy preparation processes of silica aerogels. In this work, a low-cost method was proposed to prepare silica aerogels with industrial silica hydrosol and a subsequent ambient pressure drying (APD) process. Various surfactants (cationic, amphoteric, or anionic) were added to avoid solvent exchange and surface modification during the APD process. The effects of various surfactants on the microstructure, thermal conductivity, and thermal stability of the silica aerogels were studied. The results showed that the silica aerogels prepared with a cationic or anionic surfactant have better thermal stability than that prepared with an amphoteric surfactant. After being heated at 600 °C, the silica aerogel prepared with a cationic surfactant showed the highest specific surface area of 131 m^2^∙g^−1^ and the lowest thermal conductivity of 0.038 W∙m^−1^∙K^−1^. The obtained low-cost silica aerogel with low thermal conductivity could be widely applied as a thermal insulator for building and industrial energy-saving applications.

## 1. Introduction

Silica aerogels [1,2,3,4,5,6], as a kind of highly porous material, have shown great potential application in energy-saving windows and doors [7], solar energy integration, and building energy efficiency [8,9] due to their unique structure and properties such as low density (3–500 mg·cm^−3^), high porosity (80–99.8%), low dielectric constant, and low thermal conductivity [2,10,11]. In particular, the pore size of silica aerogel is smaller than the mean free path of air molecules. This, along with its tortuous solid network, gives silica aerogel an ultra-low thermal conductivity [10,12]. Therefore, silica aerogel has promising applications in building and industrial energy saving. However, the extensive application of silica aerogel is limited by its high production cost. In general, silica aerogels are fabricated by means of the hydrolysis and condensation of silicon alkoxides to obtain wet gels and subsequent drying to remove the solvent [13,14]. Unfortunately, the raw materials (such as tetraethyl orthosilicate) used for fabrication are expensive and the supercritical drying (SCD) process is expensive and complex. To simplify the preparation process and reduce their costs, inexpensive silicon sources were adopted as precursors to prepare silica aerogels, such as silica hydrosol [15] and water glass [16,17]. To avoid the need for expensive equipment, SCD with high pressure and a long time consumption—the ambient pressure drying (APD) method developed by Smith [18]—was successfully employed to facilitate the drying process. However, the solvent exchange and surface modification involved in this APD process are still laborious, costly, and time-consuming. Moreover, ambient pressure-dried aerogels usually show serious cracking due to the fragile structure of the gels. As we know, surfactants usually can be divided into three categories: cationic, amphoteric, and anionic. The surface activity of the surfactant plays an important role in acid or alkaline solution. On the one hand, the surfactants could form a saturated adsorption layer on the surface of solvent which would reduce the surface tension of the liquid and the pore structure of the silica aerogel would be well preserved after APD. On the other hand, amphiphilic surfactant molecules could form micelles in solution with a hydrophobic core stabilized by a hydrophilic shell [19,20]. Those hydrophilic micelles would attach to the surface of silica particles and become embedded in the silica network through hydroxyl groups, which would influence the structure of the silica aerogel. After being heated at an appropriate temperature, the organic surfactants and micelles can be removed and the occupied space will be released to form more pores in the silica aerogel. Here, organic surfactants and micelles act like a “pore-forming agent”. Moreover, some recent works [21,22,23,24] on the effect of surfactants in silica aerogels have also reported.

In this work, we report a one-pot APD synthesis method to fabricate silica aerogel monoliths from industrial silica hydrosol in an aqueous system. Compared with water glass, industrial silica hydrosol is a better choice due to its relatively high purity; thus, no further ion exchange is required. In addition, there is no hydrolysis process by using silica hydrosol as a precursor and it is not necessary to accurately control the hydrolysis reaction rate. Solvent exchange and surface modification were avoided by adding various surfactants. The surfactant, which serves as a functional additive, will reduce the surface tension of the solvent and produce more nanopores after being heated at an appropriate temperature. Various silica aerogel monoliths were obtained by the addition of various surfactants under an acid–base two-step condition and subsequent APD process. The effect of types of surfactants and heat treatment on the morphology, pore structure, thermal stability, and thermal conductivity of the silica aerogels were investigated. Using a low-cost industrial silica hydrosol as the raw material and water as the solvent, along with the convenient APD process, this method greatly simplifies the preparation process and reduces the cost.

## 2. Experimental Section

### 2.1. Materials

Silica hydrosol (alkaline, 20 wt%) was purchased from Wuhan Zhifa Technology Co., Ltd. (Wuhan, China). Hydrochloric acid, ammonia hydroxide, hexadecyl trimethyl ammonium bromide (CTAB, cationic), and sodium dodecyl sulfate (SDS, anionic) were purchased from Sinopharm Chemical Reagent Co., Ltd. (Shanghai, China). Lauramidopropyl betaine (RALUFON 414, amphoteric, 35 wt%) was purchased from Shandong Yousuo Chemical Technology Co., Ltd. (Qingdao, China).

### 2.2. Preparation of Silica Aerogels with Surfactant

**Figure 1:**
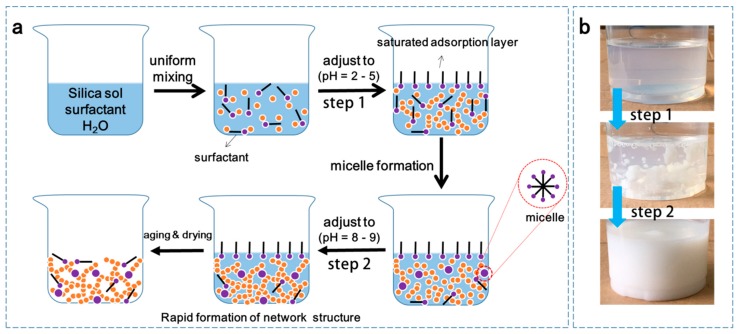
(**a**) Synthesis flowchart of silica aerogels prepared with surfactant. (**b**) Photographs of the gel evolution during the preparation of silica aerogels with surfactant.

The silica wet gels were prepared from silica hydrosol by adding the surfactant (cationic, amphoteric, or anionic) under an acid–base two-step catalytic condition, and the mixture was subsequently dried through the APD process. Firstly, 10 mL of silica hydrosol and 30 mL of water (molar ratio of SiO_2_ and water—1:44.3) were mixed together with vigorous stirring for 5 min. Then, a small amount of surfactant and hydrochloric acid solution were successively added into the mixture to keep the pH value within 2–5, and the mixture was stirred for 15 min at room temperature. Secondly, an ammonia solution was added into the mixture to adjust the pH value to 8–9, and this was further stirred for 15 min. The obtained mixture was aged in an oven at 50 °C for 4 h. Finally, the aged gel was ambient pressure-dried sequentially at 60 °C for 2 days and at 75 °C for 1 day. The optimal molar ratios of the cationic (CTAB), anionic (SDS), and amphoteric surfactant (RALUFON 414) to SiO_2_ were 1:68.7, 1:21.7, and 1:14.9, respectively. In the optimization of the amount of surfactant, the as-prepared silica aerogels have a low density, low thermal conductivity, high specific surface area, and little shrinkage. A possible synthesis flowchart of silica aerogels prepared with a surfactant is presented in Figure 1a.

Herein, the surfactant plays an important role in the construction of the network structure and APD process. For step 1, the surfactant molecules are dispersed randomly in the silica hydrosol system before the addition of the hydrochloric acid solution. When the pH value of the system is kept within 2–5, the surfactant and silica particles in the matrix will rearrange randomly to form a saturated adsorption layer and silica network structure. Because they are amphipathic, the surfactant molecules orient themselves with the hydrophobic part towards the surface and the hydrophilic part towards the aqueous phase. That is to say, the formed saturated adsorption layer can cover solvent molecules with a high surface free energy and then reduce the surface tension of the whole solution. Furthermore, when the ammonia solution is added (step 2), the surface hydrophilic surfactant micelles formed by the surfactant molecules will attach to the surface of the silica particles and connect with the silica network structure by hydroxyl groups [20], which accelerate the gelation rate and develop a new network structure. Photographs of the gel evolution during the preparation of silica aerogels with the surfactant are shown in Figure 1b. The silica cluster formed randomly in the aqueous system when a certain amount of hydrochloric acid was added. After the pH value was adjusted within 8–9, a homogeneous white wet gel was rapidly produced, as shown in the bottom of Figure 1b. It should be noted that the preparation of the whole wet gel was completed in only 30 min, indicating the fast gelation process of this method.

In order to simply distinguish silica aerogels prepared with different surfactants and heating temperatures, samples prepared with different processes were denoted using different symbols. S-1, S-2, and S-3 represent the as-prepared aerogels with CTAB (cationic), RALUFON414 (amphoteric), and SDS (anionic), respectively. S-1-200 (S-1-400 and S-1-600), S-2-200 (S-2-400 and S-2-600), and S-3-200 (S-3-400 and S-3-600) refer to silica aerogels after heat treatment at 200 °C, 400 °C, and 600 °C for 3 h, respectively.

### 2.3. Characterization

The bulk density of aerogels was determined by *ρ* = *M/V*, where *M* and *V* are the mass and volume of the aerogels, respectively. The morphology of the samples was characterized by a scanning electron microscope (SEM, S-4800). The functional groups of silica aerogels were characterized by Fourier transform infrared spectroscopy (FTIR, TENSOR 27). The weight loss of silica aerogels was tested by using an SDT-1960 universal instrument. The N_2_ adsorption–desorption isotherms of the samples were measured by a N_2_ adsorption–desorption analyzer (TriStar 3000). The specific surface areas (SSAs) and the pore size distributions were then derived from the adsorption branch using the Brunauer-Emmett-Teller (BET) method and the Barrett-Joyner-Halenda (BJH) method, respectively. The thermal conductivity was evaluated by a Hot Disk thermal constants analyzer (TPS 2500).

## 3. Results and Discussion

### 3.1. Bulk Density and Room-Temperature Thermal Conductivity

**Figure 2:**
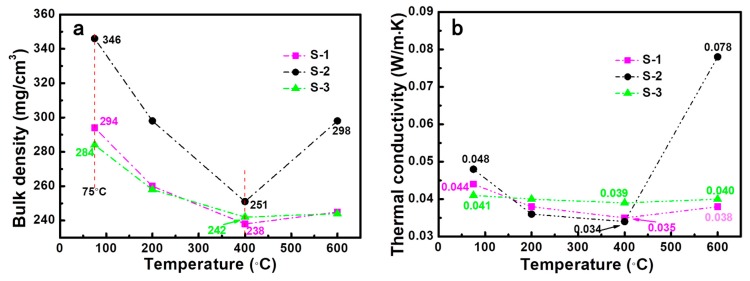
(**a**) Bulk density and (**b**) room-temperature thermal conductivity of silica aerogels before and after heat treatment at different temperatures.

The bulk density and thermal conductivity of silica aerogels heated at different temperatures are shown in Figure 2. It was observed that the bulk density and thermal conductivity were first reduced and then rose up with increasing heat treatment temperature. As shown in Figure 2a, all the as-prepared aerogels possess the highest bulk density because of the existence of organic components. The samples heated at 400 °C (S-1-400, S-2-400, and S-3-400) showed the lowest bulk densities of 238, 251, and 242 mg·cm^−3^, which was due to the elimination of organic components from the surfactant. When the heat treatment temperature further increased to 600 °C, the densification of the microstructure and collapse of the pores of aerogels normally would result in a relatively higher bulk density. We can clearly see that the density of the S-2 sample increased to 298 mg·cm^−3^ after heat treatment at 600 °C. However, the densities of S-1 and S-3 samples exhibited almost no change, which was attributed to their smaller shrinkage and better integrity.

The thermal conductivity of a material is often correlated with its density [12]. As shown in Figure 2b, the variation tendency of the thermal conductivity of the aerogels was in accordance with the change of their density levels. The lowest room-temperature thermal conductivity values of S-1, S-2, and S-3 were 0.035, 0.034, and 0.039 W·m^−1^·K^−1^ after heat treatment at 400 °C, respectively. After further heat treatment at 600 °C, the room-temperature thermal conductivity values of S-1-400 and S-3-400 showed almost no change, which was the same as the density variance. However, the thermal conductivity of S-2 increased from 0.034 to 0.078 W·m^−1^·K^−1^, which was mainly ascribed to the collapse of the skeleton and densification of the microstructure. An appropriate heat treatment temperature can effectively remove the residual organic compounds without destructing the porous structure of the aerogels. These results illustrate that the type of surfactant and heat treatment temperature have an obvious effect on the density and thermal conductivity of the silica aerogels.

### 3.2. Morphology of Silica Aerogels

**Figure 3:**
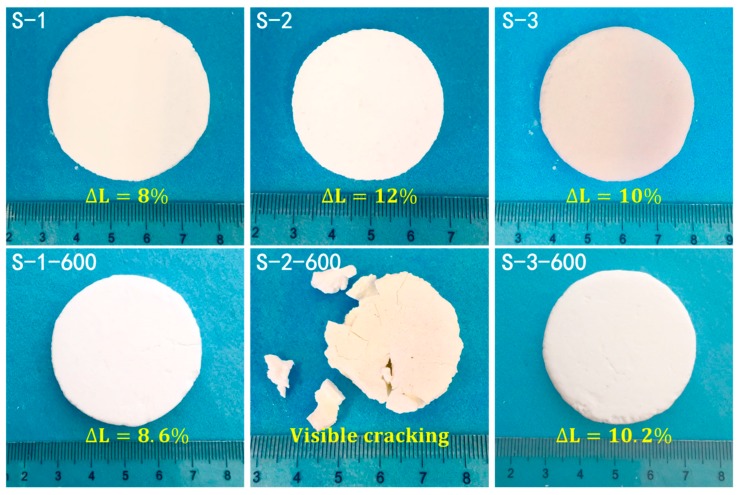
Photographs of silica aerogels prepared with different surfactants before and after heat treatment at 600 °C (∆L represents the linear shrinkage of obtained samples).

Figure 3 presents the images of silica aerogels prepared with different surfactants before and after heat treatment at 600 °C. It was observed that the color of S-1 and S-3 samples changed from beige to pure white after heat treatment at 600 °C for 3 h, which was due to decomposition of residual organic groups. An obvious cracking occurred on the S-2 sample, while S-1 and S-3 still retained a complete shape. The retention of S-1 and S-3 was derived from the effective condensation of Si-O-Si with the addition of the cationic surfactant CTAB or the anionic surfactant SDS. The effective condensation resulted in an enhancement in the network of the silica aerogels, which would also make them able to withstand high temperatures. The linear shrinkage of the monolithic silica aerogels was calculated to evaluate the thermal stability. After drying via APD, the silica aerogel monoliths prepared with all surfactants exhibited low linear shrinkage without cracking. The linear shrinkages of S-1, S-2, and S-3 were 8%, 12%, and 10%, respectively. After further heat treatment at 600 °C for 3 h, S-1 and S-3 still showed low linear shrinkage without cracking, whereas S-2 was fractured. The low shrinkage during the APD process is also beneficial for the preparation of large-sized monolithic aerogels and mass production.

**Figure 4:**
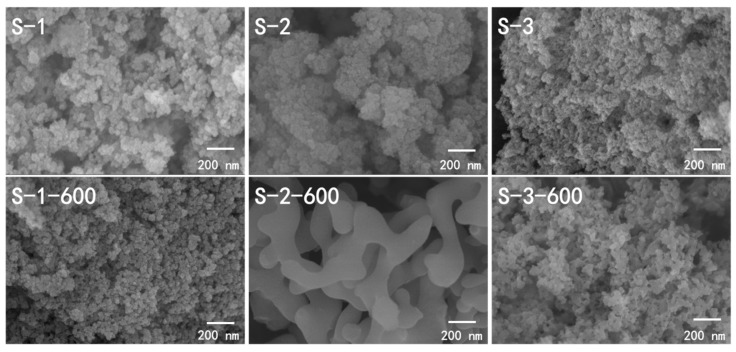
SEM images of silica aerogels prepared with different surfactants before and after heat treatment at 600 °C.

The microstructure of silica aerogels was characterized by SEM, as shown in Figure 4. There is no doubt that aerogels are highly porous solid materials with three-dimensional network structures. For the S-1 and S-3 samples, silica hydrosol can form a uniform gel network in the presence of CTAB-water or SDS-water. As expected [21], it could be seen that the presence of the surfactant impacts the microstructure of the as-prepared silica aerogels significantly. The results from Vareda and Maximiano et al. suggested that a rearrangement of the microstructure of the final material was due to the porogen effect of each surfactant in the condensation and gelation steps. As can be seen from the SEM images, S-1 and S-3 exhibited typical interconnected network structures consisting of silica particles in different sizes. However, S-2 shows a coarsened structure and observable clusters. The formation of clusters was due to the accumulation without effective interconnection of Si-O-Si in the presence of the surfactant RALUFON 414. This result indicates that the amphoteric surfactant RALUFON 414 can greatly interfere with the network formation and induce lower crosslinking among the particles due to the excessive activity in the gelation process.

The thermal stability of silica aerogels prepared with different surfactants was also evaluated by means of the change in the microstructure before and after heat treatment at 600 °C. For both the S-1-600 and S-3-600 samples, the gel network retained a uniform structure because the high-crosslinking microstructure was strong enough to withstand the high-temperature heat treatment. It was also clearly observed that the morphology of S-2 changed from a particulate structure to a coarsened and dense structure after heat treatment at 600 °C, which was caused by the low connectivity between the silica particles. The appropriate surfactant activity allows more condensation, which results in the high interconnection of silica hydrosol particles. Conversely, excess surfactant activity potentially suppresses the network formation, which leads to lower crosslinking.

### 3.3. Chemical Composition of Silica Aerogels

**Figure 5:**
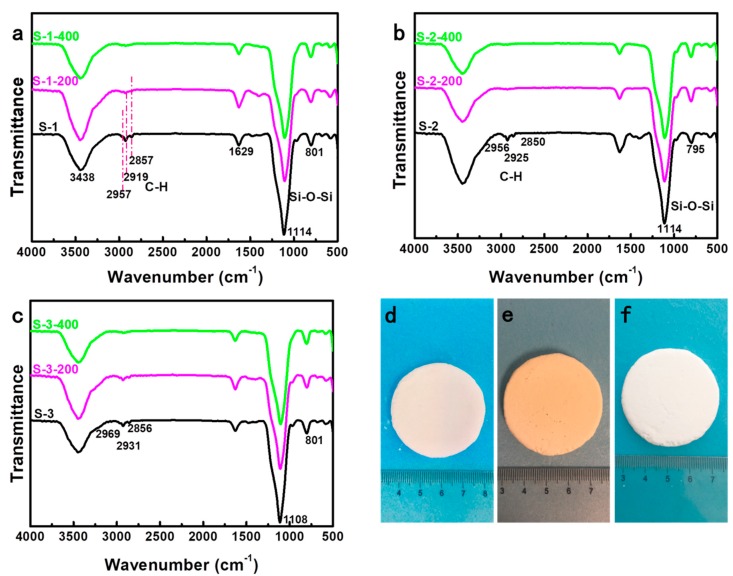
FTIR spectra of the silica aerogels: (**a**) S-1, (**b**) S-2, and (**c**) S-3 before and after heat treatment at 200 °C and 400 °C, respectively. (**d**–**f**) Representative photographs of silica aerogels prepared with sodium dodecyl sulfate (SDS) before and after heat treatment at 200 °C and 400 °C, respectively.

In order to study the chemical structure of the silica aerogels before and after heat treatment, the samples were also characterized with FTIR spectroscopy. As shown in Figure 5a–c, the spectra of S-1, S-2, and S-3 are all very similar, indicating that they feature similar chemical structures that are formed by the use of different surfactants. The broad peak near 3438 cm^−1^ and the small band near 1629 cm^−1^ correspond to the Si-OH absorption peak [25]. The absorption peak near 1114 cm^−1^ is due to the asymmetrical stretch vibration of Si-O-Si, while the peak at 801 cm^−1^ is the result of the symmetrical stretching vibration of Si-O-Si [25,26,27,28]. Moreover, the absorption peaks around 2800–3000 cm^−1^ are assigned to the stretching vibrations of C-H [25,26,29]. After heat treatment at 200 °C for 3 h, the absorption peaks near 2800–3000 cm^−1^ decrease, and almost disappear after further heat treatment at 400 °C for 3 h, indicating the decomposition of organic compositions from the surfactants.

A set of representative photographs of silica aerogels prepared with SDS are shown in Figure 5d–f. The color of the silica aerogels changed from beige to brown after heat treatment at 200 °C, and then to pure white after heat treatment at 400 °C. This color evolution also demonstrates that the organic compositions in silica aerogels gradually decomposed with the increase of the heat treatment temperature, which agrees with the results of the FTIR spectra.

### 3.4. Thermogravimetric Analysis (TGA) of Silica Aerogels

**Figure 6:**
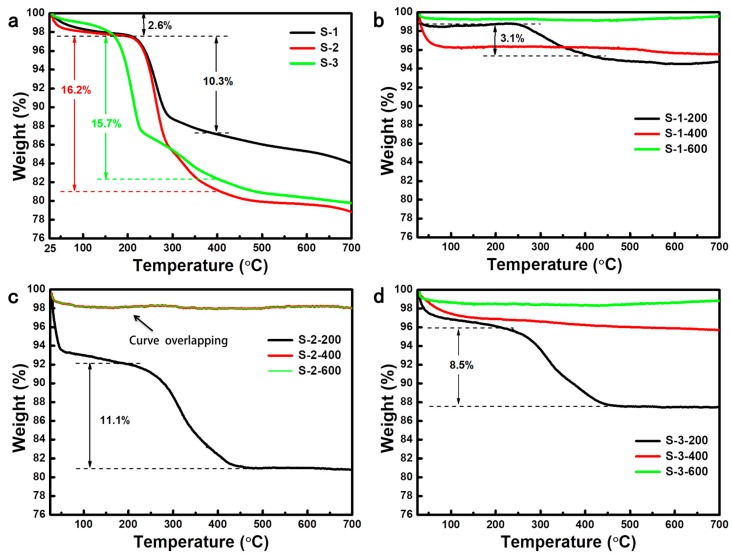
Weight loss curves of the silica aerogels: (**a**) S-1, S-2, and S-3; (**b**) S-1-200, S-1-400, and S-1-600; (**c**) S-2-200, S-2-400, and S-2-600, the curve of S-2-600 is almost overlapped with that of S-2-400; (**d**) S-3-200, S-3-400, and S-3-600.

Figure 6 shows the weight loss curves of silica aerogels prepared with various surfactants before and after heated at different temperatures. As exhibited in Figure 6a, the weight loss of S-1, S-2, and S-3 could be divided into three regions: 25~200 °C, 200~400 °C, and 400~700 °C. The first region with a small weight loss of 2.6% was caused by the evaporation of the solvent, the second region was attributed to the decomposition of organic components, and the third region was due to the dehydration of Si-OH. As presented in Figure 6a, S-1 and S-2 have a higher organic component decomposition temperature (about 220 °C) than S-3 (about 170 °C), which indicates the better thermal stability of CTAB and RALUFON 414 as compared to SDS. The weight loss curves of silica aerogels prepared with various surfactants after heated at different temperatures are shown in Figure 6b–d. It is observed that there are still a lot of weight losses between 200 and 400 °C for silica aerogels after heat treatment at 200 °C due to the decomposition of residual organic components. Neglect of the small weight loss between 25 and 100 °C caused by the evaporation of the adsorbed water, there are almost no weight changes of silica aerogels after heat treatment at 400 and 600 °C. These results confirm that organic components from surfactants in all samples were removed completely after being heated at 400 °C, which is consistent with the FTIR results.

### 3.5. Pore Structures of Silica Aerogels

**Figure 7:**
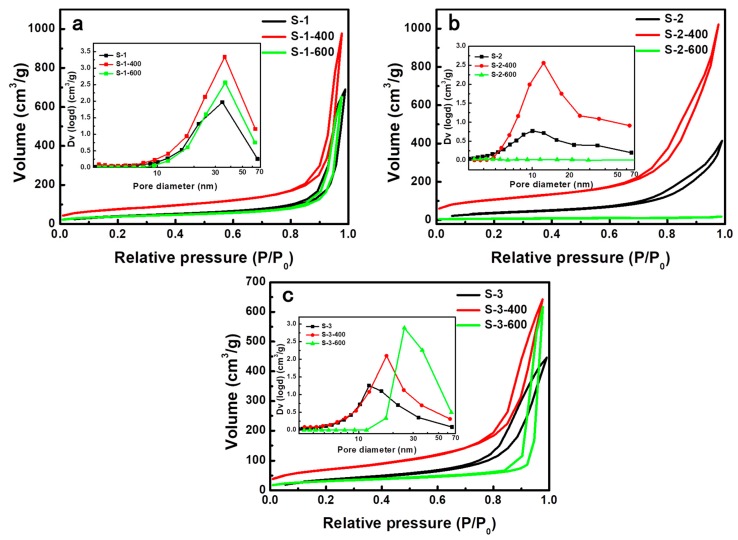
N_2_ adsorption–desorption isotherms and pore-size distributions of silica aerogels: (**a**) S-1, (**b**) S-2 and (**c**) S-3 before and after heat treatment at 400 °C and 600 °C, respectively.

The N_2_ adsorption-desorption isotherms and pore size distributions of silica aerogels prepared with various surfactants are shown in Figure 7. All the isotherms of silica aerogels represent a Type IV isotherm with an H3 type hysteresis loop, which is the characteristic of mesoporous materials [30,31]. The detailed pore structure parameters are presented in Table 1. After APD, the specific surface areas of the silica aerogels prepared with various surfactants were almost the same, ranging from 134 m^2^·g^−1^ to 141 m^2^·g^−1^. As shown in the inset pore-size distribution in Figure 7 and Table 1, the silica aerogel prepared with CTAB (S-1) had larger average pore size (24.7 nm) and larger pore volume (1.07 cm^3^·g^−1^) than the silica aerogels prepared with RALUFON 414 (S-2) and SDS (S-3), respectively. After being heated at 400 °C, the specific surface areas of all silica aerogels largely increased due to the removal of organic surfactants and the opening of more pores. As mentioned above, the amphiphilic surfactant molecules would form micelles in the solution with a hydrophobic core stabilized by a hydrophilic shell, and those hydrophilic micelles will attach to the surface of silica particles and embed in the silica network through hydroxyl groups. These act as a “pore forming agent” after being removed by heat treatment. The removal of organic surfactants was also confirmed by the FTIR spectra in Figure 5. It is noted that the silica aerogel prepared with RALUFON 414 (S-2) possessed a much higher SSA (378 m^2^·g^−1^) than S-1 (264 m^2^·g^−1^) and S-3 (247 m^2^·g^−1^) after being heated at 400 °C. However, the nano-pore structure of S-2 was collapsed and its network was coarsened after being heated at 600 °C, as shown in Figure 4, which led to a significant loss of SSA. After heating at 600 °C, the SSA of S-2 dramatically decreased to 22 m^2^·g^−1^ and the pore volume accordingly decreased to 0.02 cm^3^·g^−1^. By contrast, the silica aerogels S-1 and S-3 maintained high SSAs of 131 and 107 m^2^·g^−1^, respectively, which indicates that they had better thermal stability than S-2. Combined with the thermal conductivities exhibited in Figure 2b, the silica aerogel prepared with the amphoteric surfactant (S-2) possessed a higher SSA and lower thermal conductivity than the silica aerogels prepared with the cationic surfactant (S-1) and anionic surfactant (S-3) after being heated at 400 °C. The silica aerogels prepared with the cationic surfactant (S-1) and anionic surfactant (S-3) had better thermal stability than the silica aerogel prepared with the amphoteric surfactant (S-2).

## 4. Conclusions

In summary, low-cost silica aerogels were prepared with industrial silica hydrosol and a subsequent ambient pressure drying (APD) process. Solvent exchange and surface modification during the APD process were avoided by the addition of various surfactants (cationic, amphoteric, and anionic). The pore structure of the silica aerogels was well preserved after APD thanks to the addition of surfactants that reduced the surface tension of the liquid. Moreover, organic surfactants and micelles were removed to release more pores after the mixture was heated at 400 °C. The silica aerogel prepared with the amphoteric surfactant (RALUFON 414) possesses a higher SSA (378 m^2^·g^−1^) and lower thermal conductivity (0.034 W·m^−1^·K^−1^) than the silica aerogels prepared with the cationic (CTAB) and anionic surfactants (SDS) after heat treatment at 400 °C. However, the silica aerogels prepared with the cationic (CTAB) and anionic surfactants (SDS) exhibited a better thermal stability than that prepared with the amphoteric surfactant (RALUFON 414). After being heated at 600 °C, the silica aerogel prepared with CTAB maintained a high SSA of 131 m^2^·g^−1^ and a low thermal conductivity of 0.038 W·m^−1^·K^−1^, respectively. This low-cost and easily mass-produced silica aerogel with a high SSA, low thermal conductivity, and good thermal stability could be widely applied as a thermal insulator for building and industrial energy-saving applications.

## Figures and Tables

**Table 1:** Pore structures of silica aerogels prepared with various surfactants before and after heat treatment at 400 °C and 600 °C, respectively.

Sample	Specific Surface Area (SSA) (m^2^·g^−1^)	Average Pore Size (nm)	Pore Volume (cm^3^·g^−1^)
S-1	141	24.7	1.07
S-1-400	264	35.7	1.49
S-1-600	131	36.1	1.02
S-2	141	13	0.64
S-2-400	378	9.6	1.6
S-2-600	22	3.3	0.02
S-3	134	14	0.69
S-3-400	247	17.5	0.99
S-3-600	107	25.1	0.99
